# Extensive hyperpigmentation during pregnancy: a case report

**DOI:** 10.1186/1752-1947-5-464

**Published:** 2011-09-19

**Authors:** Anthony Massinde, Salvatore Ntubika, Moke Magoma

**Affiliations:** 1 Department of Obstetrics and Gynecology, Bugando Medical Centre, Box 1370, Mwanza, Tanzania; 2 Department of Internal Medicine/Dermatology, Bugando Medical Centre, Box 1370, Mwanza, Tanzania

## Abstract

**Introduction:**

Skin hyperpigmentation is common during pregnancy and often is due to endocrinological changes. Usual patterns include linea nigra, darkening of areola and melasma. We report a rare diffused hyperpigmentation condition in a pregnant woman of dark colored skin.

**Case presentation:**

A 19-year-old Tanzanian primigravida at 32 weeks gestation presented at our antenatal clinic concerned about an insidious but progressive onset of unusual darkening of her abdominal skin and both breasts. Her antenatal record was unremarkable except for this unusual onset of abnormal skin color. Findings from her physical examination were unremarkable, and she had a normal blood pressure of 120/70 mmHg. Her abdomen was distended with a uterine fundus of 34 weeks. Almost her entire abdominal skin had darkly colored diffuse deep hyperpigmentation extending cephalad from both iliac fossae to involve both breasts to 2-3 cm beyond the areolae circumferentially. She had a fetus in longitudinal lie and cephalic presentation, with a normal fetal heart rate of 140 beats per minute. Other examination findings were unremarkable. The impression at this stage was exaggerated pigmentation of pregnancy. No medical treatment was offered but she was counseled that she might need medical treatment after delivery. She progressed well and had spontaneous labor and normal delivery at 38 weeks gestation. She was lost to follow up.

**Conclusion:**

Unusual pregnancy-related skin hyperpigmentation can occur with no adverse consequences to pregnancy, although may worry a pregnant woman. Reassurance and conservative management may be all that is required to allay a patient's concerns.

## Introduction

Hyperpigmentation during pregnancy is commonly due to endocrinological changes. The usual pattern will be seen as linea nigra, melasma and darkening of areola, axillae and medial thighs [1-3]. Extensive hyperpigmentation, however, is unusual, especially in people with dark colored skin [4,5]. Such hyperpigmentation may sometimes be associated with hyperthyroidism [1,5]. We present a case of an unusual pattern of pigmentation in a primigravida seen in her mid-third trimester, who had an unremarkable pregnancy, labor, delivery and postpartum period.

## Case presentation

A 19-year-old Tanzanian primigravida at 32 weeks of gestation sought care at a tertiary hospital antenatal clinic. She presented with concerns of an insidious but progressive onset of an unusual darkening of her abdominal skin and both breasts. The darkening was not associated with itching or irritation of the skin.

She booked for antenatal care at a peripheral clinic and her progress had been unremarkable except for this unusual onset of abnormal skin color. She had no previous history of allergies or family history of skin condition. She was not on any medication except for prescribed iron and folic acid tablets given during antenatal consultations. Her past medical history was unremarkable with no history suggestive of goiter or hyperthyroidism.

**Figure 1:**
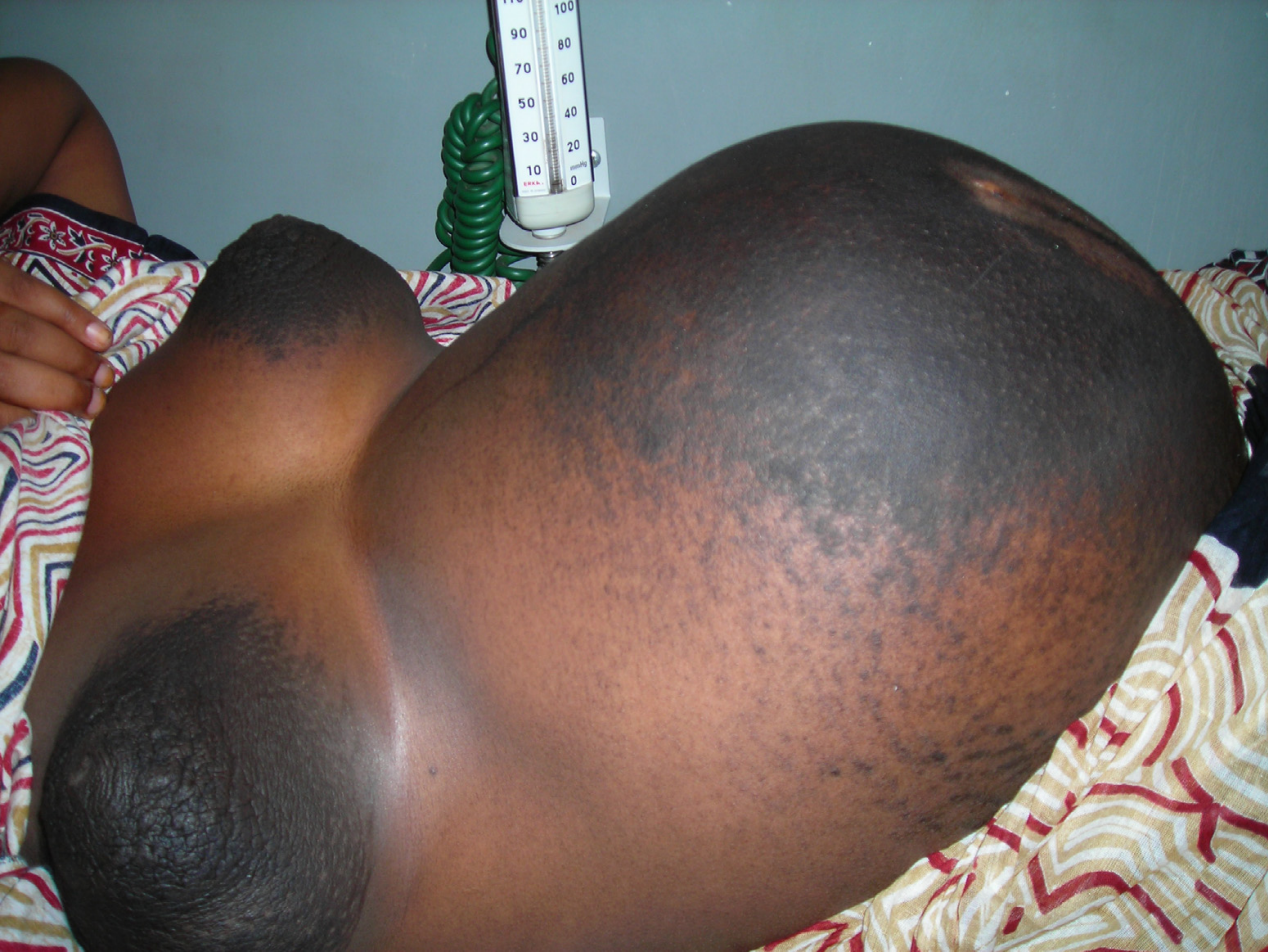
**Exaggerated hyperpigmentation of pregnancy**. Extensive hyperpigmentation of pregnancy involving both breast and abdominal skin.

On physical examination, her general condition was fair. She was not pale and had no lower limb edema. She had a pulse rate of 70 beats per minute that was regular. Her blood pressure was 120/70 mmHg. Her abdomen was distended with a uterine fundus of 34 weeks. A linea nigra was clearly seen, but in addition almost the entire abdominal skin had dark colored diffuse deep hyperpigmentation, extending from both iliac fossae to involve both breasts (nipples and areolae) to about 2-3 cm beyond the areolae circumferentially (Figure 1). She had a fetus in longitudinal lie, cephalic presentation with a normal fetal heart rate of 140 beats per minute. Other system examination findings were unremarkable.

The impression at this stage was exaggerated pigmentation of pregnancy. No medications were prescribed, but she was reassured that the condition should have no effect on her pregnancy and its outcome. She continued attending antenatal care regularly. She had spontaneous onset of labor and normal delivery of a baby girl weighing 3200 g with an Apgar score of 9 and 10 at the first and fifth minutes respectively at 38 weeks gestation.

Our patient did not return for follow-up during her postpartum period. She was contacted by phone three months after delivery and she reported that her skin condition had not resolved. We lost contact with her thereafter.

## Discussion

Skin hyperpigmentation is common in pregnancy and often is well described and completely benign in nature [2,5]. The physiology of hyperpigmentation appears to be related to the increased production of estrogens, and perhaps to increased levels of progesterone or a melanocyte-stimulating hormone [1-3,5]. In selected areas of the body such as the linea alba and areola, hyperpigmentation is probably related to the distribution of melanocytes, but extension of these cells beyond these parts may explain unusual patterns of distribution, as in this case [2,3,6].

The intensity of the hyperpigmentation, however, may be related to environmental factors or even intake of some drugs, although other causes may include pre-existing conditions, such as hyperthyroidism, and a genetic predisposition [1,3,5]. Nevoid hyperkeratosis of the nipple and areola should be considered in the absence of abdominal involvement. Dermal melanocytosis is another rare condition that could present similarly to our case; in this condition pregnancy and sun-exposure are thought to be the triggering factors [5].

Most pregnancy-related skin hyperpigmentation is benign and is usually resolved after delivery (usually within a year), although women may be concerned [1,2,5]. Medical treatment is rarely required. In cases where the condition persists, bleaching agents may be used [1,3,5], although at times their effectiveness may be unsatisfactory [5]. Proper counseling and assurance is the only reliable alternative in such cases [5].

## Conclusion

Although skin hyperpigmentation is common in pregnancy, extensive pigmentation, as in this case, is rare. Patients may be cosmetically concerned, but all that is required from the health professional is reassurance that the condition has no adverse affect on pregnancy outcome.

## Consent

Written informed consent was obtained from the patient for publication of this case report and accompanying image. A copy of the written consent is available for review by the Editor-in-Chief of this journal.

## Competing interests

The authors declare that they have no competing interests.

## Authors' contributions

AM managed the patient and wrote the initial manuscript. ST performed the initial literature search. Both ST and MM reviewed the subsequent manuscripts and approved the final manuscript.

## References

[B1] Wade TR Wade SL Jones HE Skin changes and diseases associated with pregnancy Obstet Gynecol 1978 52 2 233 242 683665

[B2] Elling SV Powell FC Physiological changes in the skin during pregnancy Clin Dermatol 1997 15 1 35 43 10.1016/S0738-081X(96)00108-3 9034654

[B3] Blereau RP Three cases of hyperpigmentation of pregnancy Consultantlive 2002 42 10

[B4] Tunzi M Gray GR Common skin conditions during pregnancy Am Fam Physician 2007 75 2 211 218 17263216

[B5] Ingber A Lebwohl M hyperpigmentation and melasma Obstetric Dermatology 2009 Jerusalem: Springer 7 17

[B6] Szabo G The number of melanocytes in human epidermis Br Med J 1954 1 4869 1016 1017 10.1136/bmj.1.4869.1016 13149891 PMC2084967

